# Radiotherapy for localized sebaceous carcinoma of the eyelid: a retrospective analysis of 83 patients

**DOI:** 10.1093/jrr/rrz046

**Published:** 2019-07-04

**Authors:** Yoshiaki Takagawa, Wakana Tamaki, Shigenobu Suzuki, Koji Inaba, Naoya Murakami, Kana Takahashi, Hiroshi Igaki, Yuko Nakayama, Naoyuki Shigematsu, Jun Itami

**Affiliations:** 1 Department of Radiation Oncology, National Cancer Center Hospital, Tokyo, Japan; 2 Department of Radiation Oncology, Prefectural Chubu Hospital, Okinawa, Japan; 3 Department of Ophthalmology, National Cancer Center Hospital, Tokyo, Japan; 4 Department of Radiology, Keio University School of Medicine, Tokyo, Japan

**Keywords:** radiotherapy, sebaceous carcinoma, eyelid, prognostic factor, toxicity

## Abstract

The current study retrospectively analyzed the results of radiotherapy for clinically localized sebaceous carcinoma of the eyelid.We reviewed records of 83 patients with histologically confirmed sebaceous carcinoma who were treated radiotherapeutically between 1983 and 2015. Sixty-five patients (78%) were initially treated with radiotherapy of curative intent, while the remaining 18 patients underwent postoperative radiotherapy due to tumor recurrence or positive surgical margins. Thirty-seven patients belonged to T1–2, while 46 belonged to T3–4. All 83 patients were treated with radiotherapy with a median radiation dose of 60 Gy. The median follow-up period was 92.1 months (range, 2.8–310.3 months).

At the time of analysis, 13 patients (15.1%) died, and 36 patients (43.3%) had local recurrence. The 7-year overall survival, freedom from neck lymph node recurrence, and local control (LC) rates for all patients were 83.5%, 75.5%, and 52.3%, respectively. Patients with a tumor size ≤10 mm had a higher 7-year LC rate than those with a tumor size >10 mm (58.8% vs 46.6%, *P* = 0.054). Neck lymph node recurrence was observed in 17 patients (20%) and significantly related to the tumor size. Late toxicity of an eyelid dysfunction of grade 3 was observed in 1 patient with T3 tumor.

Radiotherapy for sebaceous carcinoma of the eyelid is a reasonable alternative to surgical resection for tumors <10 mm in size with few severe complications, while larger tumors should be treated with surgery if feasible.

## INTRODUCTION

**Fig. 1.**
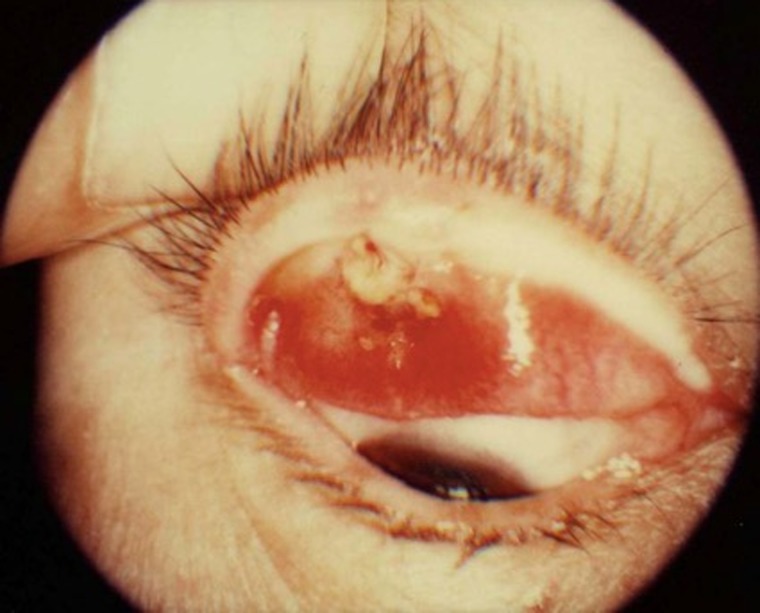
Typical finding of sebaceous carcinoma of the eyelid.

Sebaceous carcinoma of the eyelid is an uncommon neoplasm that accounts for <1% of all eyelid tumors and approximately 5% of all eyelid malignancies [1, 2]. This tumor develops most commonly among Asian women of around 70 years of age. Sebaceous carcinoma most commonly arises from the Meibomian glands anterior to the gray line and occasionally from the glands of Zeis or Moll and sebaceous glands in the caruncle [3]. Typical finding of sebaceous carcinoma of the eyelid is shown in Figure 1. All too frequently, sebaceous carcinoma is misdiagnosed as chalazion, blepharoconjunctivitis, and basal or squamous cell carcinoma. Through the direct invasion of adjacent organs, such as the eyeball and brain, and distant metastasis in the advanced stages, sebaceous carcinoma results in a disease-related mortality rate of 6−30% [4, 5]. Accordingly, early diagnosis and treatment are imperative.

Radical surgical excision with a frozen section control by either a standard method or Mohs micrographic surgery is the most common and effective treatment method of sebaceous carcinoma. However, due to advanced age, presence of coexisting diseases, or refusal of surgery, some patients are unsuitable for surgery. Furthermore, despite a recent progress in reconstructive surgery, eyelid tumors may be difficult to excise completely without functional and cosmetic impairment [6, 7]. Therefore, radiotherapy is a treatment option for patients who refuse or are otherwise unsuitable for surgery. However, limited information is currently available on the role of radiotherapy in treating these tumors.

In the present study, we retrospectively analyzed the efficacy and safety of radiotherapy for the treatment of clinically localized sebaceous carcinoma of the eyelid.

## MATERIALS AND METHODS

### Patient characteristics

A retrospective review of medical records in our institution from 1983 to 2015 identified 89 patients with a histologically confirmed sebaceous carcinoma of the eyelid. Four patients who had lymph node metastases and/or distant metastases at the time of diagnosis were excluded. Additionally, one patient with a diagnosis of hereditary retinoblastoma was excluded because of prior ocular irradiation with a total dose of 40 Gy, and another patient who was irradiated palliatively with only 30 Gy in 15 fractions was also excluded. The remaining 83 patients with clinically localized tumors were included in this analysis. All patients were treated with radiotherapy with curative intent. Patient and treatment characteristics are shown in Table 1. There were 37 men and 46 women. The patients’ age ranged from 28 to 94 years with a median of 67 years, and their Eastern Cooperative Oncology Group performance status ranged from 0 to 3 (median, 1). The maximum tumor size ranged from 4 to 35 mm (median, 12 mm), while a tumor size <10 mm was noted in 25 patients. Epithelial invasion was identified in 34 patients (41%).

**Table 1.** Patient and treatment characteristics

Total patients	83
Female/male	46 (55%)/37 (45%)
Median age/range	67 years/28–94 years
Performance status 0–1/2–3	76/7
Indication for radiation therapy	
Definitive therapy as an initial treatment	65 (78%)
Postoperative salvage therapy for recurrent tumor	11 (13%)
Postoperative adjuvant therapy for positive surgical margins	7 (9%)
Tumor location	
Upper eyelid	50 (60%)
Lower eyelid	30 (36%)
Upper and lower eyelid	3 (4%)
Maximum tumor size	
Median/range	12 mm/4–35 mm
T stage (UICC 6th)	
T1 (≤5 mm)	7 (9%)
T2 (5–10 mm)	30 (36%)
T3 (>10 mm)	38 (45%)
T4 (invades adjacent structures)	8 (10%)
Radiation dose (EQD_2 Gy_) (α/β = 10)	
<60 Gy	9 (11%)
60 Gy	64 (77%)
>60 Gy	10 (12%)
Radiation modality, *n*(%)	
Electron	77 (93%)
Photon	6 (7%)
Bolus	35 (42%)
Lead-based lens block	68 (82%)

EQD_2 Gy_ = equivalent dose in 2 Gy.

Sixty-five patients (78%) were initially treated with radiotherapy, while the remaining 18 patients (22%) initially underwent surgical resection. The principle of treatment of the eyelid sebaceous carcinoma was considered to be surgery for any T stage, however, if there was a serious systemic disease, large plastic surgery was required, or for refusal of surgery, radiotherapy was used as an initial treatment. Of the 18 patients who underwent surgical resection, 11 patients underwent salvage radiotherapy for postoperative recurrent tumors, while 7 patients were treated with postoperative adjuvant radiotherapy for positive surgical margins. In the current study, we used the TNM staging system of the Union Internationale Contre le Cancer (UICC) 6th edition [8]. In patients undergoing postoperative salvage radiotherapy, T stage was allocated to the recurrent tumor size, while patients undergoing adjuvant radiotherapy were classified by the preoperative tumor status. Seven patients had a T1 tumor, 30 patients had a T2 tumor, 38 patients had a T3 tumor, and 8 patients had a T4 tumor.

### Treatments

All 83 patients were treated with radiotherapy with curative intent. The treatment volume was determined based on physical examinations, pretreatment computed tomography (CT), or magnetic resonance imaging (MRI) scans, and the planning target volume (PTV) included at least 5-mm margins from the primary tumor. Radiotherapy was administered with 3–12 MeV electron appositional field in 77 patients (93%), and 4 MV X-rays were used in 6 patients (7%). X-ray irradiation was delivered with a single anterior portal in 3 patients and two anterior angled portals in the remaining 3 patients. Two patients undergoing X-ray irradiation had T4 tumor, one had invasion of striated muscles of the orbit, and another had invasion of the nasal bone and bulbar conjunctiva. During radiotherapy, a bolus was used in 35 patients (42.2%). Thickness of the bolus was 5 mm in 15 patients, 10 mm in 1 patient, and records of the bolus thickness were lacking in the remaining 19 patients. A lead-based lens block was inserted between the eyelid and eyeball with local anesthetics and lubricant in 68 patients (81.9%). In the remaining patients, the eye was fixed by gazing at an object in the contralateral side to exclude the lens from the direct radiation beam. The dose of electron beam irradiation was prescribed to the peak dose point of the beam central axis, and the electron energy was selected to enclose the PTV with at least 80% of the peak dose. In X-ray irradiation, the dose was prescribed to the isocenter. Daily fractional dose of 1.8–2.0 Gy, administered 5 days per week, was used in 77 of 83 patients (92.8%), while daily fractional dose of 2.2–2.5 Gy was used in the remaining 6 patients. The total dose for the primary tumor in all patients ranged from 48.0 to 70.4 Gy (median, 60.0 Gy). To compare various radiotherapies with different fractional doses, the equivalent dose in a fractional dose of 2 Gy (EQD_2 Gy_) was calculated according to the Linear-Quadratic (LQ) model assuming a/b = 10 Gy.

### Statistical analysis

The median follow-up period for all 83 patients was 92.1 months (range, 2.8–310.3 months). Overall survival (OS), freedom from neck lymph node recurrence (FFNR), and local control (LC) rates were calculated according to the Kaplan−Meier method [9], starting from the initiation of radiotherapy. In the univariate analysis, statistical differences were estimated using the log-rank test [10]. Multivariate analyses for LC, FFNR, and OS were performed by Cox proportional hazards model using potential prognostic factors employed in the univariate analysis. A probability level <0.05 indicated statistical significance. Statistical analysis was performed using the SPSS software (version 23.0; IBM, Armonk, NY, USA). Late toxicities were graded according to the CTCAE version 4.0 [11]. This retrospective study was approved by the institutional review board.

## RESULTS

**Fig. 2.**
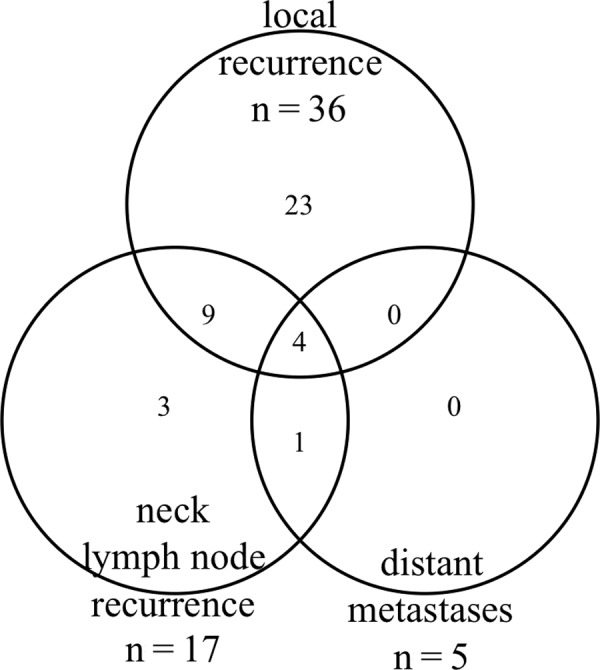
Recurrence pattern of 83 patients with sebaceous carcinoma after radiotherapy.

At the time of this analysis, 36 patients (43.3%) had disease recurrences. Twenty-three patients had only local recurrence, 3 had only neck lymph node recurrence, 13 had both local and neck lymph node recurrences, 1 had neck lymph node recurrence and distant metastases, and 4 had local and neck lymph node recurrences and distant metastases (Figure 2). Therefore, local recurrence was noted in 36 patients (43.3%), and neck lymph node recurrence in 17 patients (20%).

**Fig. 3.**
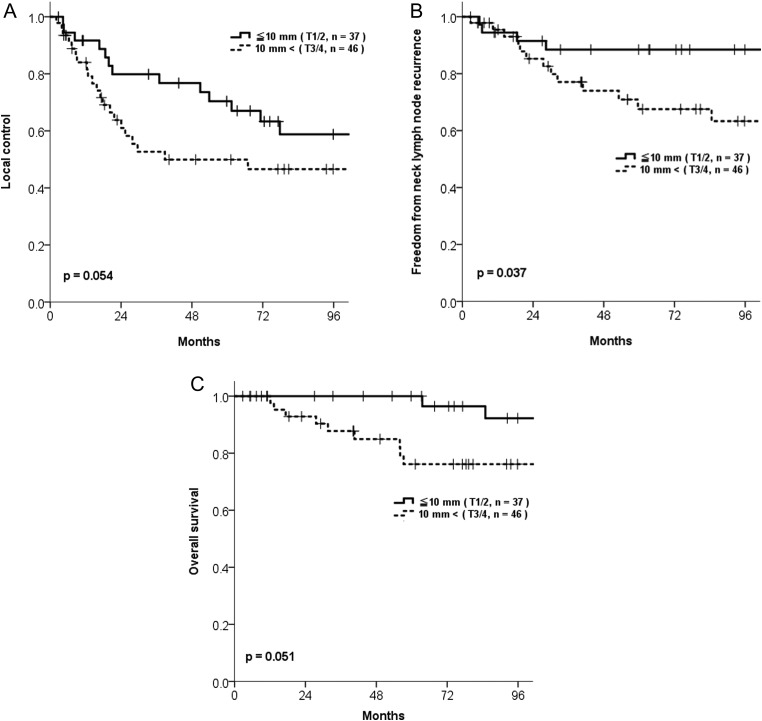
Outcomes according to tumor size and T stage in patients with sebaceous carcinoma of the eyelid. **(A)** Local control. **(B)** Freedom from neck lymph node recurrence (FFNR). **(C)** Overall survival.

The 7-year LC rate of all 83 patients was 52.3% [95% confidence interval (CI) :0.40–0.64]. Patients with a tumor size ≤10 mm had a higher 7-year LC rate than those with a tumor size >10 mm (58.8% vs 46.6%, *P* = 0.054) (Table 2, Fig. 3). If the analysis was confined to patients undergoing radiotherapy of ≥60 Gy in EQD_2 Gy_, 7-year LC rates of T1–2 (33 patients) and T3–4 (41 patients) were 60.0% and 44.2%, respectively, with an almost statistically significant difference (*P* = 0.053). Six patients had a late local recurrence more than 5 years after radiation therapy and only local recurrence without lymph node and distant metastases. Three of them were treated with local resection, and the other 3 patients were treated with exenteration of the eyelid and orbital contents, with all 6 patients successfully salvaged without further recurrence. We also analyzed the LC rate of patients undergoing postoperative salvage radiotherapy for recurrent tumor and those undergoing postoperative adjuvant radiotherapy for positive surgical margins. Although the 7-year LC rate in postoperative adjuvant radiotherapy group was favorable compared with those of postoperative salvage radiotherapy group (71.4% vs 51.9%, respectively; *P* = 0.736), a statistically significant difference in LC was not shown because 1 patient in the postoperative adjuvant radiotherapy group had local recurrence 9 years after radiotherapy. In the multivariate analysis, tumor size and radiation dose did not have a statistically significant impact on LC (Table 2).

**Table 2.** Univariate and multivariate analyses of various potential prognostic factors for LC, FFNR and OS in patients with sebaceous carcinoma of the eyelid treated with radiotherapy

	No.	7-year LC				7-year FFNR				7-year OS			
		UVA		MVA		UVA		MVA		UVA		MVA	
		Rate (%)	*P* value	HR (95 %CI)	*P* value	Rate (%)	*P* value	HR (95 %CI)	*P* value	Rate (%)	*P* value	HR (95 %CI)	*P* value
PS			0.439	−	−		0.925	−	−		0.629	2.861	0.059
0–1	76	53.8				75.5				84.3		(0.959–8.537)	
2–3	7	34.3				80.0				66.7			
Sex			0.141	−	−		0.201	−	−		0.065	0.431	0.217
Female	46	57.7				81.8				89.5		(0.113–1.638)	
Male	37	45.2				67.5				76.5			
Age			0.577	−	−		0.475	−	−		0.012	1.251	0.010
<70	49	56.5				77.6				90.5		(1.055–1.484)	
≥70	34	45.4				73.3				71.7			
T stage (UICC 6th)			0.232	0.972	0.948		0.134	1.821	0.305		0.224	2.033	0.200
T1 (≤5 mm)	7	66.7		(0.415–2.274)		100		(0.579–5.729)		100		(0.686–6.020)	
T2 (5–10 mm)	30	56.0				85.8				89.8			
T3 (>10 mm)	38	44.1				66.5				74.2			
T4	8	56.3				35.0				87.5			
Tumor size			0.054	1.031	0.332		0.037	1.014	0.758		0.051	1.148	0.286
≤10 mm	37	58.8		(0.969–1.098)		88.4		(0.928–1.108)		92.2		(0.891–1.481)	
>10 mm (including T4)	46	46.6				63.3				76.1			
RT dose (EQD_2 Gy_)			0.537	0.930	0.882		0.926	1.943	0.524		0.506	3.941	0.300
≥60 Gy	73	52.4		(0.353–2.445)		74.7		(0.252–14.976)		83.0		(0.294–52.768)	
<60 Gy	10	50.0				80.0				87.5			
Indication for RT			0.719	1.142	0.583		0.310	1.156	0.658		0.521	0.662	0.493
Initial treatment	65	51.1		(0.712–1.831)		77.9		(0.608–2.197)		86.5		(0.204–2.151)	
Salvage RT	11	51.9				77.1				76.2			
Adjuvant RT	7	71.4				51.4				68.6			

EQD_2 Gy_ = equivalent dose in 2 Gy, FFNR = freedom from neck lymph node recurrence, HR = hazard ratio, LC = local control, MVA = multivariate analysis, OS = overall survival, PS = performance status, RT = radiation therapy, UVA = univariate analysis.

The 7-year FFNR rate of all 83 patients was 75.5% (95%CI: 0.65–0.86). The relatively high rate of neck lymph node recurrence (17 of 83 patients, 20%) prompted us to investigate the risk factors for its recurrence. Table 2 shows the FFNR rates according to various potential risk factors. In the univariate analysis, patients with a tumor size ≤10 mm had a significantly higher 7-year FFNR rate than those with a larger tumor size (88.4% and 63.3%, respectively; *P* = 0.037; Table 2, Fig. 3). However, in the multivariate analysis, tumor size did not have a statistically significance effect on FFNR (Table 2).

Of 83 patients, 13 (15.1%) died during the follow-up period. Of these 13 patients, 4 died of sebaceous carcinoma, and the remaining 9 died without clinical recurrence: lung cancer (*n* = 3) and pancreatic cancer (*n* = 1) were the causes of death, and five patients died of unknown cause (*n* = 5). The 7-year OS rate for all patients was 83.5% (95%CI: 0.74–0.93), and age had a significant impact on OS in the univariate and multivariate analyses (Table 2).

Table 3 outlines the late toxicities in all patients. Grade 3 or more late toxicities were observed in only 1 patient (grade 3 eyelid dysfunction). This patient had T3 tumor that was treated with radiotherapy with a total dose of 60 Gy. Grade 2 cataract developed in 1 patient. Twenty-five patients had cataract already before radiation therapy due to old age. We could not find any relationship between lead-based lens block and late toxicities. Moreover, approximately 10–30% of patients had grade 1–2 late toxicities including watery eye, dry eye, eyelid dysfunction, and keratitis.

**Table 3.** Late toxicities (*n* = 83)^a^

	No. of patients	Grade		
		1	2	3
Cataract	1 (1.2%)	0	1	0
Dry eye	8 (9.6%)	6	2	0
Watery eye	11 (13.2%)	3	8	0
Keratitis	15 (18.1%)	11	4	0
Eyelid dysfunction	29 (34.9%)	14	14	1

^a^ No grade 4 or 5 toxicities were observed.

## DISCUSSION

This is the largest study on radiotherapy of sebaceous carcinoma of the eyelid, which includes 83 patients. Surgical resection has been considered as the standard treatment of sebaceous carcinoma of the eyelid. However, the current study revealed that radiotherapy yields comparable LC in sebaceous carcinoma with a tumor size ≤10 mm (58.8% at 7 years). Although the proportions of patients with early-stage tumors were not documented, several studies have reported the efficacy of radiotherapy in LC of sebaceous carcinoma [12, 13]. Hata *et al.* treated 5 patients with radical radiotherapy for gross tumors with a median maximum diameter of 12 mm and found that all 5 patients achieved LC [13]. Pardo *et al.* analyzed 4 patients treated with radical radiotherapy (total doses of 45–63 Gy) and found that no patients had recurrences at the primary tumor sites during the follow-up period of 60–17 months [12]. After wide local excision or Mohs micrographic surgery, sebaceous carcinoma of the eyelid can be controlled locally in 60–90% [14–17]. Erovic *et al.* analyzed 33 patients with periorbital sebaceous carcinomas (76% were classified as T1 or T2) treated with primary surgery. The 5-year LC rate was 63%, and the 5-year regional control rate was 58% [17]. The radiotherapeutic results of sebaceous carcinoma with a tumor size ≤10 mm in the current study seemed to be comparable to the surgical series. However, LC rate in the current study was lower than that in the recent series on Mohs micrographic surgery. Previous reports on Mohs micrographic surgery had a small sample size and shorter follow-up period than the current study [15]. Sebaceous carcinoma of the eyelid can recur locally very late even after 5 years as stated. Therefore, long-term follow-up is also needed in the surgery reports.

Moreover, the current study indicated that radiotherapy achieved a 7-year LC rate of 46.6% even for sebaceous carcinoma of the eyelid with a larger tumor size (>10 mm, including T4). Several authors emphasized that a tumor size >10 mm and extensive invasion (represented as T3-4 tumors) are closely related to the risk of recurrence and metastases [18]. This study revealed that radiotherapy can be a viable alternative to surgery even in the patients with T3-4 tumors. In our study, patients with T4 tumor had a higher 7-year LC rate than those with T2 and T3 tumors (56.3%, 56.0%, and 44.1%, respectively; Table 2), for which we could not determine the reason. However, a favorable LC rate of T4 tumors might be related to the small tumor size <20 mm in 5 of 7 patients with T4 tumors.

The optimal dose for radiotherapy with curative intent for sebaceous carcinoma of the eyelid remains unknown. Nunery *et al.* found that all 6 patients with sebaceous carcinoma of the eyelid experienced local recurrence with radical radiotherapy at total doses of 33–54 Gy (median, 50 Gy) in 1.8–3.1 Gy fraction size [19]. Hendley *et al.* treated 3 patients with sebaceous carcinoma of the eyelid with radiotherapy at 48 Gy and observed local recurrence in a single patient [20]. In contrast, Hata *et al.* conducted definitive radiotherapy with total doses of 60–66 Gy in 5 patients with sebaceous carcinoma of the eyelid with fraction sizes of 1.8–2 Gy in 4 patients and 3 Gy in 1 patient. The authors found no recurrences within the radiation field in any of the treated patients [13]. In the current study, a 7-year LC rate of 60.0% was obtained in patients with T1-2 tumors treated with ≥60 Gy. The results suggest that a dose of ≥60 Gy in conventional fractionation may be insufficient to eradicate even T1-2 tumors. The 7-year LC rate in a postoperative adjuvant radiotherapy group was favorable compared with those of a postoperative salvage radiotherapy group and initial definitive radiotherapy group (71.4% vs 51.9%, 51.1%). In the postoperative adjuvant radiotherapy group, 3 of 7 patients had local recurrence. One patient treated with 50 Gy recurred in 108.9 months after radiotherapy. The other 2 patients treated with 60 Gy recurred in 6.3 months and 14.3 months after radiotherapy, respectively. While the remaining 4 patients treated with >60 Gy in EQD_2Gy_ (1 patient was 59.4 Gy in 27 fractions, 2 patients were 60 Gy in 30 fractions and 1 patient was 66 Gy in 33 fractions) achieved LC. Therefore, we consider that a dose of ≤60 Gy was insufficient to eradicate even in a postoperative adjuvant radiotherapy setting. The bolus was used only in 35 of 77 patients who underwent electron beam therapy. Without bolus, the surface dose is lower, especially in 3-MeV electron irradiation. Additionally, PTV was enclosed by at least 80% of the prescription dose in electron irradiation. Therefore, the dose to PTV appear be lower than the prescribed dose in some patients. Further optimization of electron beam irradiation might improve the LC rate of sebaceous carcinoma.

The optimal radiation field for sebaceous carcinoma of the eyelid is also unknown. When performing surgery, several authors recommended that surgical margins of 5–6 mm are required to completely excise tumors at a microscopic level [6, 21]. For example, Dogru *et al.* reported that recurrence occurred in the primary lesion in 36% of patients who underwent surgical resection with 1–3-mm margins from the edge of gross tumors but that no local recurrence was observed in patients with at least 5-mm margins [21]. Hata *et al.* treated 5 patients with gross tumors by radiotherapy with 10-mm margins and found that no patients had local recurrences [13]. In the current study, we could not obtain adequate information regarding the patterns of local failure, such as in-field, marginal, or out-field recurrence. However, considering the previous reports, the gross tumor with at least 5-mm margins appears to be preferable for clinical target volume when conducting radiotherapy.

Sebaceous carcinoma of the eyelid has often been reported to lead to neck lymph node recurrence, particularly in the preauricular, parotid, or submandibular lymph nodes in 8−32% of patients [4, 5]. In agreement with the previous series, 20% of patients treated with radical radiotherapy in the current study developed neck lymph node recurrence. It remains unknown whether prophylactic irradiation to the neck lymph nodes is necessary for patients with clinically N0 tumors. Our results indicated that tumor size was a significant risk factor for FFNR in the univariate analysis. Esmaeli *et al.* reported that T stage was significantly associated with lymph node metastasis in surgery cases [22]. To our knowledge, the current study is the first report to indicate the risk factors for neck lymph node recurrence in patients treated with radiotherapy. The 7-year FFNR rate was 88.4% in patients with a tumor size ≤10 mm but only 63.3% in patients with a larger tumor size. Additionally, of 17 patients with neck lymph node recurrences, 13 patients had both local and neck lymph node recurrences. Ten of 13 patients had local recurrence before or concurrent with neck lymph node recurrence (Figure 2). This suggests that improvement in LC could favorably affect FFNR. However, prophylactic neck irradiation appears to be warranted in patients with a tumor size >10 mm, including T4 tumors. A total dose of 45–50 Gy appears to be appropriate for prophylactic neck irradiation in patients with N0 diseases. Indeed, Hata *et al.* performed prophylactic neck irradiation with doses of 44–50.5 Gy and reported no neck lymph node recurrences within the radiation field [13].

Recently, systemic chemotherapy with various agents, such as doxorubicin, cisplatin, and 5-fluorouracil, is reported to have achieved a significant reduction in the volume of bulky eyelid tumors and contributed to prolonged survival [23]. Therefore, multimodal treatment, including surgery and radiotherapy with or without chemotherapy, may be appropriate for T3-4 tumors.

In the current study, late morbidity of grade 3 or more was observed in only 1 patient, suggesting that radiotherapy in the treatment of these tumors is quite safe. Only 1 patient developed radiation-induced cataract. Several authors have also indicated that radical radiotherapy is associated with few severe late toxicities [1]. In the current study, approximately 10–30% of patients experienced grade 1–2 late toxicities, including watery eye, dry eye, eyelid dysfunction, and keratitis with a median total dose of 60 Gy. These results indicate that radiotherapy is associated with few grade 3 or higher late toxicities, while some patients may have grade 1–2 late toxicities. However, the true incidence of radiation-induced cataract might be underestimated because cataract had already been present before radiation therapy in 25 patients due to old age. Lead shields may be effective in reducing late toxicities, but we could not find the correlation between lead shields and late toxicities. Because of the retrospective nature of this study and the study period extending to more than 30 years, some patients were lost to follow-up, and the incidence of late toxicities may be underestimated.

## CONCLUSION

Radiotherapy for sebaceous carcinoma of the eyelid is a reasonable alternative to surgical resection for tumors <10 mm in size with few severe complications, while larger tumors should be treated with surgery with or without radiotherapy, if feasible. Radiotherapy alone can be a reasonable alternative even for patients with T3–4 tumors (>10 mm) if surgical removal is not possible. Prophylactic neck irradiation appears to be required for patients with T3-4 tumors. Further investigations are required to determine the optimal radiation dose and field for radiotherapy of sebaceous carcinoma.
